# Transcriptional and Mycolic Acid Profiling in *Mycobacterium bovis* BCG In Vitro Show an Effect for c-di-GMP and Overlap Between Dormancy and Biofilms

**DOI:** 10.4014/jmb.1911.11043

**Published:** 2020-03-13

**Authors:** Miguel A. De la Cruz, Miguel A. Ares, Diana Rodríguez-Valverde, Alba Adriana Vallejo-Cardona, Mario Alberto Flores-Valdez, Iris Denisse Cota Núñez, Michel de Jesús Aceves-Sánchez, Jonahtan Lira-Chávez, Jacobo Rodríguez-Campos, Jorge Bravo-Madrigal

**Affiliations:** 1 Unidad de Investigación Médica en Enfermedades Infecciosas y Parasitarias, Centro Médico Nacional (CMN) Siglo XXI, Instituto Mexicano de Seguro Social (IMSS), Ciudad de México, México; 2 Centro de Investigación y Asistencia en Tecnología y diseño del Estado de Jalisco (CIATEJ) A.C., Biotecnología Médica y Farmacéutica, Av. Normalistas No. 800. Colinas de la Normal, C.P. 4470 Guadalajara, Jalisco, México; 3 Centro de Investigación y Asistencia en Tecnología y diseño del Estado de Jalisco (CIATEJ) A.C, Unidad de Servicios Analíticos y Metrológicos, Av. Normalistas No. 800. Colinas de la Normal, C.P. 44270 Guadalajara, Jalisco, México

**Keywords:** Tuberculosis, BCG, Mycolic acids, c-di-GMP, transcriptional profiling, biofilms

## Abstract

*Mycobacterium tuberculosis* produces mycolic acids which are relevant for persistence, recalcitrance to antibiotics and defiance to host immunity. c-di-GMP is a second messenger involved in transition from planktonic cells to biofilms, whose levels are controlled by diguanylate cyclases (DGC) and phosphodiesterases (PDE). The transcriptional regulator *dosR*, is involved in response to low oxygen, a condition likely happening to a subset of cells within biofilms. Here, we found that in *M. bovis* BCG, expression of both *BCG1416c* and *BCG1419c* genes, which code for a DGC and a PDE, respectively, decreased in both stationary phase and during biofilm production. The *kasA*, *kasB*, and* fas* genes, which are involved in mycolic acid biosynthesis, were induced in biofilm cultures, as was *dosR*, therefore suggesting an inverse correlation in their expression compared with that of genes involved in c-di-GMP metabolism. The relative abundance within trehalose dimycolate (TDM) of α- mycolates decreased during biofilm maturation, with methoxy mycolates increasing over time, and keto species remaining practically stable. Moreover, addition of synthetic c-di-GMP to mid-log phase BCG cultures reduced methoxy mycolates, increased keto species and practically did not affect α-mycolates, showing a differential effect of c-di-GMP on keto- and methoxy-mycolic acid metabolism.

## Introduction

Tuberculosis (TB) remains a major public health problem worldwide and is the leading cause of death due to a single pathogen. According to the World Health Organization, in 2016 there were an estimated 10.4 million new TB cases across the world with 1.7 million deaths (WHO, 2017). Furthermore, about one-quarter of the world´s population is infected with *Mycobacterium tuberculosis* in a dormant stage, meaning that 2 billion people have latent TB infection (LTBI) [1]. People living with LTBI are asymptomatic and do not transmit the disease, but they face the risk of developing active TB [2]. On the other hand, during in vitro dormancy, *M. tuberculosis* establishes a non-replicating persistence (NRP) state, with adaptation to microaerophilic conditions and later hypoxia, where the transcriptional regulator DosR and its regulon play a key role in adapting to this state [3]. DosR has been shown to be fundamental for persistence of *M. tuberculosis* in non-human primates [4]. Regarding mycobacterial persistence in vivo, it has been proposed that biofilms might give rise to adaptations similar to those necessary for *M. tuberculosis* during chronic TB infection [5]. It is likely that bacteria grown as surface pellicles or aggregates termed biofilms might face different availability of nutrients and oxygen, depending on where within the aggregates they are residing, and therefore *dosR* might be activated in those cells with limited access to oxygen, although this remains to be experimentally evaluated.

The intracellular second messenger molecule 3´, 5´-cyclic diguanylic acid (c-di-GMP) controls the transition from planktonic to biofilm stages in many bacteria. c-di-GMP is synthesized from two GTP molecules by diguanylate cyclases (DGC) and is degraded by phosphodiesterases (PDE) [6]. The *Rv1354c* and *Rv1357c* genes of *M. tuberculosis* code for a diguanylate cyclase and a phosphodiesterase, respectively; although Rv1354c is also able to degrade c-di-GMP in vitro [7]. *Mycobacterium bovis* BCG contains homologs to *Rv1354c* and Rv1357c, the *BCG1416c* and *BCG1419c* genes, respectively, and the contribution of *BCG1419c* to surface pellicle and biofilm attached to the plastic surface of tissue culture flasks has already been reported [8]. On the other hand, several studies have revealed the important role of mycolic acids (MA) in the development of mycobacterial biofilms since these lipids are the most abundant molecules in the cell wall of *Mycobacteria* [9-11]. As we previously reported by transcriptomic analysis the diminished expression of some genes involved in MA biosynthesis, such as *acpM, kasA, kasB*, and *fas* in the BCGΔBCG1419c mutant strain with respect to wild-type BCG [12], this suggested a link between the levels of the mycobacterial second messenger c-di-GMP and metabolism of MAs, specifically, as the mutant in the PDE-encoding gene is predicted to produce more c-di-GMP than parental BCG, this implies that this second messenger might have a negative role for *acpM, kasA, kasB*, and *fas* transcription during biofilm production by BCG.

It is known that *M. tuberculosis* H37Rv produces mature biofilms after 4-5 weeks of growth in Sauton media [9], but no information has been reported as to how this strain progresses or controls biofilm production in a time-course manner. Hence, in this work, we have characterized the production of surface pellicles and attached biofilms in a time-course manner in the *M. tuberculosis* complex bacteria, *Mycobacterium bovis* BCG, Pasteur strain, and analyzed the transcriptional expression of genes implicated in c-di-GMP and MA metabolism during in vitro growth of planktonic and sessile cells in *M. bovis* BCG. We went further to determine how the MA profiles vary at three time points during biofilm maturation. Finally, we evaluated *dosR* transcription because we hypothesize that low oxygen might be found by mycobacteria during biofilm production, at least for some part of the population.

Our data show that transcription of genes involved in MA synthesis varies as the biofilm maturates and that there are specific modifications in the relative presence of α, keto- and methoxy-MAs, which when coupled to experiments where synthetic c-di-GMP was added to mid-log phase BCG cultures, show a differential effect of cdi- GMP on keto- and methoxy-mycolic acid metabolism.

## Materials and Methods

### Bacterial Strains and Culture Conditions

The *M. bovis* BCG Pasteur strain was obtained from the American Type Culture Collection (*Mycobacterium bovis* Karlson and Lessel (ATCC 35734), Strain Designations: TMC 1011 [BCG Pasteur] ), and it was grown in Middlebrook 7H9 (broth) or 7H10 (agar) media supplemented with 0.2%/0.5% glycerol, respectively, plus 10%OADC, and 0.05% Tween 80 (only for 7H9). Aerobic growth was achieved by culturing mycobacterial strains in 7H9 OADC broth plus 0.05% Tween 80, at 37°C, 100 rpm, in an orbital shaker, using 75 cm^2^ vented cap tissue culture flasks (BD Falcon). For planktonic cultures, samples were taken at OD600nm 0.4 (log phase) and 1.2 (stationary phase) for qPCR determinations, and at OD600nm 0.6 for determining the effect of synthetic c-di-GMP (Sigma, sodium salt, SML1228), where cultures were either added or not to this second messenger and further incubated for 6 h before harvest and extraction with petroleum ether. For surface pellicle and biofilm formation, BCG was grown in Sauton media (containing asparagine 4.0 g/l, sodium citrate 2.0 g/l, K_2_HPO_4_ 0.5 g/l, MgSO_4_ 0.5 g/l, ferric ammonium citrate 0.05 g/l, glycerol 0.06%, ZnSO_4_ 0.001 g/l, glucose 4.82 g/l, pH 7.0–7.4) and samples of the whole surface pellicle along with bacteria attached to the plastic wall of the flasks (hereafter referred to as biofilms) were taken at days 7, 10, and 14 of culture. Two independent experiments, with biological duplicates for each one, were used for photographs of surface pellicles, total RNA and lipids isolation, except for surface pellicle and biofilm conditions as described below.

### Biofilm Formation

BCG Pasteur was cultured in 20 ml of Middlebrook 7H9 OADC 0.2% glycerol plus 0.05% Tween 80 until stationary phase (OD_600 nm_ = 1.2). Then, 6 ml of bacterial culture was homogenized in 230 ml of detergent-free Sauton medium, and the initial OD_600nm_ was 0.034. Later, 10 different aliquots of 20 ml from the above subculture were transferred into 25cm^2^ tissue culture flask with vented cap, 0.2 μM-filtered. All of these were incubated at 37°C in a 5% CO_2_ incubator. After 1, 7, 10, and 14 days of incubation, two culture flasks were photographed to record surface pellicle and biofilm formation. We spent around 1 min per photograph and placed the flasks back into the incubator. After completing the imaging, we removed again the flasks from the incubator and the whole biofilm was removed with a scraper. Because of the low number of cells present in the first day of culture (24 h samples), we transferred the uppermost layer by pipetting it from the flask to a 50 ml tube. Then we centrifuged this at 3,500 ×*g* for 10 min, and the pellet was stored at -70ºC. On the other hand, the whole surface pellicle and attached biofilms from 7 to 14 days were directly harvested with a scraper and transferred into 50 ml tubes that were immediately frozen at -70°C. Two biological replicates for days 7, 10, and 14 were used, whereas four replicates were used for the 24 h culture. After completing the kinetics of biofilm production in CIATEJ, we either lyophilized samples and shipped them to CMNSXXI-IMSS for RNA isolation and qPCR or used them for lipid analyses performed at CIATEJ as described below.

### RNA Isolation and Quantitative RT-PCR (qRT-PCR)

Total RNA was isolated from the different culture conditions using the hot phenol method [13] with some modifications. Briefly, cells were harvested by centrifugation and pellets were resuspended in 500 μl of lysis buffer (0.5% SDS, 20 mM sodium acetate and 10 mM EDTA). Cells were lysed mechanically in a Tissuelyser II (Qiagen, USA) with 150-212 μM glass beads (Sigma, USA) by performing three cycles of 1 min each at high speed (30 Hz).

After the lysate was obtained, and equal volume of acid-saturated phenol was added, mixed and incubated at 65°C during 10 min. The samples were chilled on ice and centrifugated at 19,000 ×*g* for 10 min at 4°C. The aqueous layer was transferred to a new tube; RNA was precipitated and washed with cold absolute ethanol and cold 70% ethanol, respectively. After careful removal of ethanol, RNAs were air-dried for 15 min in a Centrifugal Vacuum Concentrator 5301 (Eppendorf). The RNA pellets were resuspended in DEPC-treated water (Invitrogen, USA). Purification of RNA from contaminating genomic DNA was performed using the TURBO DNA-free Kit (Ambion). RNA integrity was analyzed in a bleach 2% agarose gel [14] and quantified by spectrophotometry using a Nanodrop ND-1000 (Thermo Scientific, USA). cDNA was synthesized using 500 ng of RNA, 0.2 μg/μl of random hexamer primers and 2 U/μl of RevertAid M-MuLV-RT (reverse transcriptase of Moloney Murine Leukemia Virus; Thermo Scientific). Specific gene primers were designed with the Primer3Plus software and are listed in Table 1. The absence of contaminating DNA was controlled by lack of amplification products after 35 qPCR cycles using RNA as template. Control reactions with no template (water) and with no reverse transcriptase were run in all experiments, with no transcript detected. Quantitative real-time PCR was performed in a LightCycler 480 instrument (Roche) and 16S rRNA (*rrs*) was used as a reference gene for normalization. The relative gene expression was calculated using the 2^-ΔΔCt^ method [15]. The transcription of *sigA* (principal sigma factor) was also measured as expression control.

### Heatmap Construction

In order to illustrate the fold changes in gene expression we used the heatmapper web server (http://www.heatmapper.ca/expression/) according to the relative gene expression obtained by comparing with respect to the different control conditions.

### TDM Extraction and Methylation of Fatty and Mycolic Acids

We carried out extraction of trehalose dimycolate (TDM) as previously reported [16]. For this, 7, 10, and 14 day-old cultures were harvested into pre-weighed glass tubes, centrifuged to a pellet, washed with PBS and centrifuged again at 2,500 ×*g* for 10 min to remove the media. Extraction was conducted 3 times using petroleum ether in a 1:3 (W/V) ratio. The extract was combined and dried and the tubes were weighed again to calculate the amount of TDM recovered, to then solubilize this in chloroform: methanol (2:1) for analysis by thin layer chromatography (TLC), followed by UHPLC-ESI-QTOF.

We used 30-40 mg of dried petroleum ether-extracted lipids to perform methylation of fatty acids and MAs according to [17], using 2 ml of tetrabutyl ammonium hydroxide (TBAH) and incubation overnight at 100°C. Methyl esterification of the fatty acids/MAs was conducted by adding to the TBAH mixture, 4 ml of CH_2_Cl_2_, 300 μl of CH_3_I and 2 ml of water. We incubated this for 1 h at room temperature on a rotator, followed by centrifugation at 3,500 ×*g* for 10 min at room temperature to recover the organic phase containing the lipids, which were dried under a stream of nitrogen using a TurboVap evaporator. To the dried residue, we added 3 ml of diethyl ether and sonicated this in a water bath for 10 min at room temperature. Then, we centrifuged this at 3,500 rpm for 10 min at room temperature and transferred the fatty acid/MA methyl esters into a new glass tube, to evaporate the diethyl ether under a stream of nitrogen and resuspend the residue (FAMEs and MAMEs) in 100-200 μl CH_2_Cl_2_.

For TLC, we used 300 μg of the FAMEs and MAMEs obtained from each strain and dissolved them in chloroform-methanol (2:1) at a ratio of 100 μl of solvent to 300 mg of wet weight. Separation was done in Silica gel 60 F_254_ plates (5 × 7 cm, Merck) by using petroleum ether: acetone (95:5) run three times. The plates were air-dried, immersed into a phosphomolybdic acid solution, air-dried again, followed by charring at 120°C for 5 min. TLC plates were visualized by UV and by spraying with phosphomolybdic acid solution. These spots were quantitatively assessed using a digital image system. All reagents for lipid extraction or modification were HPLC-grade.

### Chromatography and Mass Spectrometry Characterization

Chromatographic analysis was performed in a Waters Acquity UHPLC Class H Xevo 62-XS Qtof (Waters Corporation, USA). Separation was performed on an ACQUITY UPLC BEH Amide 1.7 μm column, and the flow rate was 0.3 ml/min. The mobile phase consisted of A (ammonium acetate 10 mM, pH9), and B (0.1% formic acid in 95% acetonitrile). The linear elution gradient program was used as follows: 0-2 min, 90% (**A**)-10% (**B**); 2-3 min, 70% (**A**)-30% (**B**); 3-5 min, 50% (**A**)-50% (**B**); 5-6 min, 100% B; 6-10 min, 100% B; 11 min 99% (**A**)-1% (**B**). UHPLC was followed by an electrospray ion source operating in positive ESI mode. The conditions of analysis were as follows: ESI, positive mode, capillary voltage of 3.0 kV, sampling cone voltage was 30.0 V. Source temperature was 100°C; the desolvation gas temperature was 500°C, and desolvation gas flow was 700 l/h. The sample infusion flow rate was 5 μl/min, with scan time of 1 s. The full-scan MS data were produced across the m/z range of 50-2,500 Da. Chromatograms were combined to generate combined spectra and a corresponding spectrum list using MassLynx 4.0 (Waters Corp. USA). Trehalose 6,6′-dimycolate from *Mycobacterium bovis* (Sigma T3034, not shown) was used as control in TLC and UHPLC-ESI-QTOF determinations.

### Statistical Analysis

For statistical differences in qPCR assays, a two-tailed unpaired *t*-test was performed using Prism 7.0 (GraphPad Software Inc, USA) for the relative expression of every single gene at 7, 10, and 14 days of the surface pellicle/biofilm culture as compared to stationary phase planktonic cultures; *p* ≤ 0.05 was considered statistically significant.

## Results

### Planktonic BCG Differentially Regulates Expression of Genes Involved in Metabolism of c-di-GMP and Mycolic Acids as Well as *dosR*

**Fig. 1:**
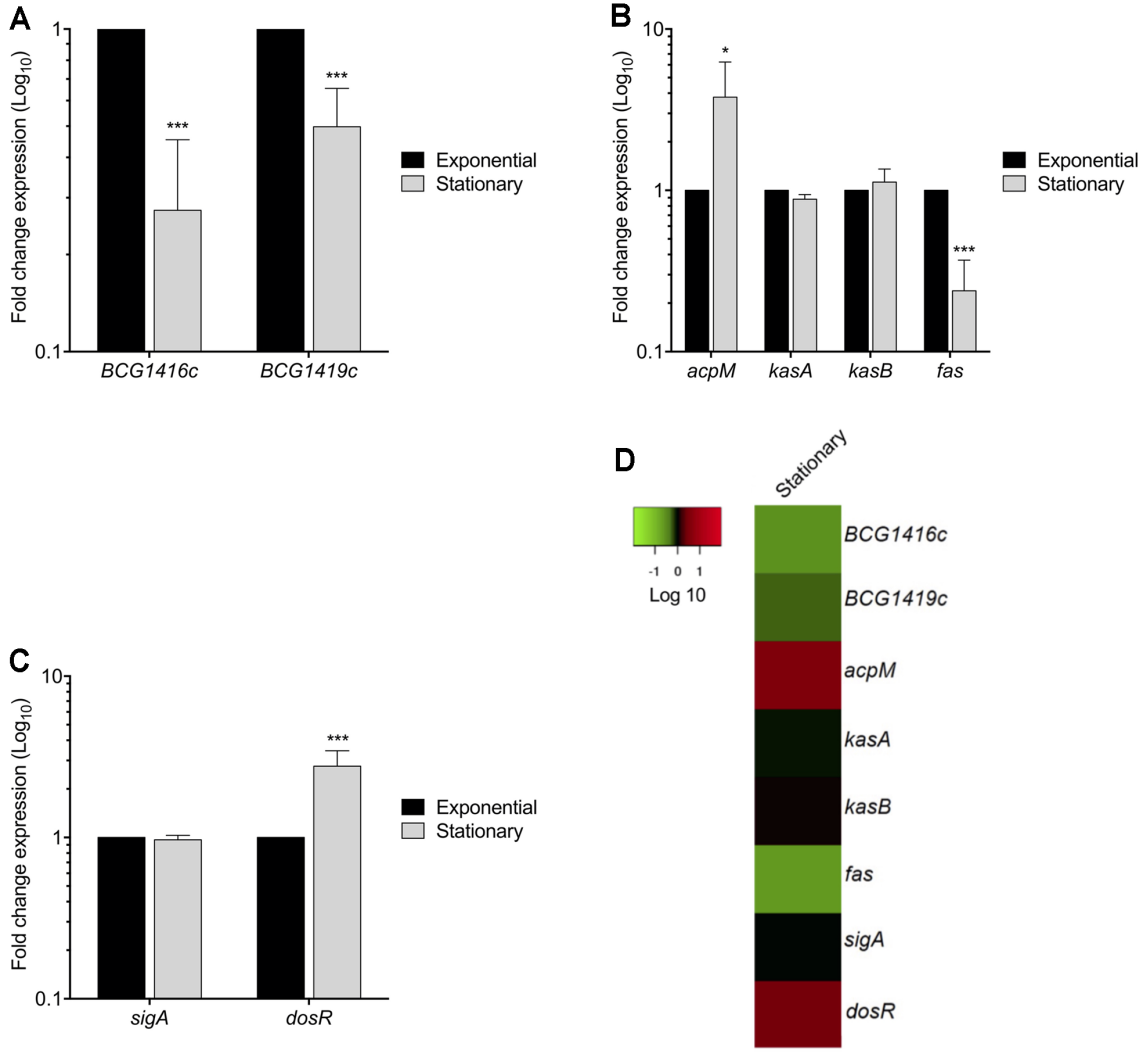
Expression of c-di-GMP, *dosR*, and mycolic acid metabolism-related genes of *M. bovis* BCG during its in vitro growth. (**A**) Fold change expression (qRT-PCR) of *BCG1416c* and *BCG1419c* genes, (**B**) *acpM, kasA, kasB* and *fas* genes, (**C**) *sigA* and *dosR* genes. (**D**) Heatmap of gene expression levels expressed as fold change in a Log_10_ scale of stationary phase relative to exponential phase. The color-coding scale shows up-regulation in red and down-regulation in green. Data represent the mean of two independent experiments performed in triplicates (mean ± SD). 16S rRNA was used as a reference gene for normalization. Statistically significant was considered as follows; ns, not significant, *p < 0.05, ***p < 0.001.

In order to determine the transcriptional expression levels of genes involved in c-di-GMP and MA metabolism during planktonic growth, as well as *dosR*, we performed qRT-PCR experiments during both exponential and stationary growth phases. We found that the expression of *BCG1416c* and *BCG1419c* diminished 3.6- and 2.0-fold in stationary phase compared to exponential phase, respectively (Figs. 1A and D). On the other hand, MA biosynthesis genes were differentially expressed: the expression of *acpM* and *fas* increased and decreased 3.8- and 4.2-fold in stationary phase, respectively, while *kasA* and *kasB* were similar in both exponential and stationary phases (Figs. 1B and D). The transcription of *sigA* was similar in both exponential and stationary phases, while *dosR* transcription increased 2.8-fold in stationary phase (Figs. 1C and D), in agreement with previously reported data [18, 19].

### Temporal Profiling of Genes Involved in c-di-GMP, *dosR*, and Mycolic Acid Metabolism during Biofilm Formation

**Fig. 2:**
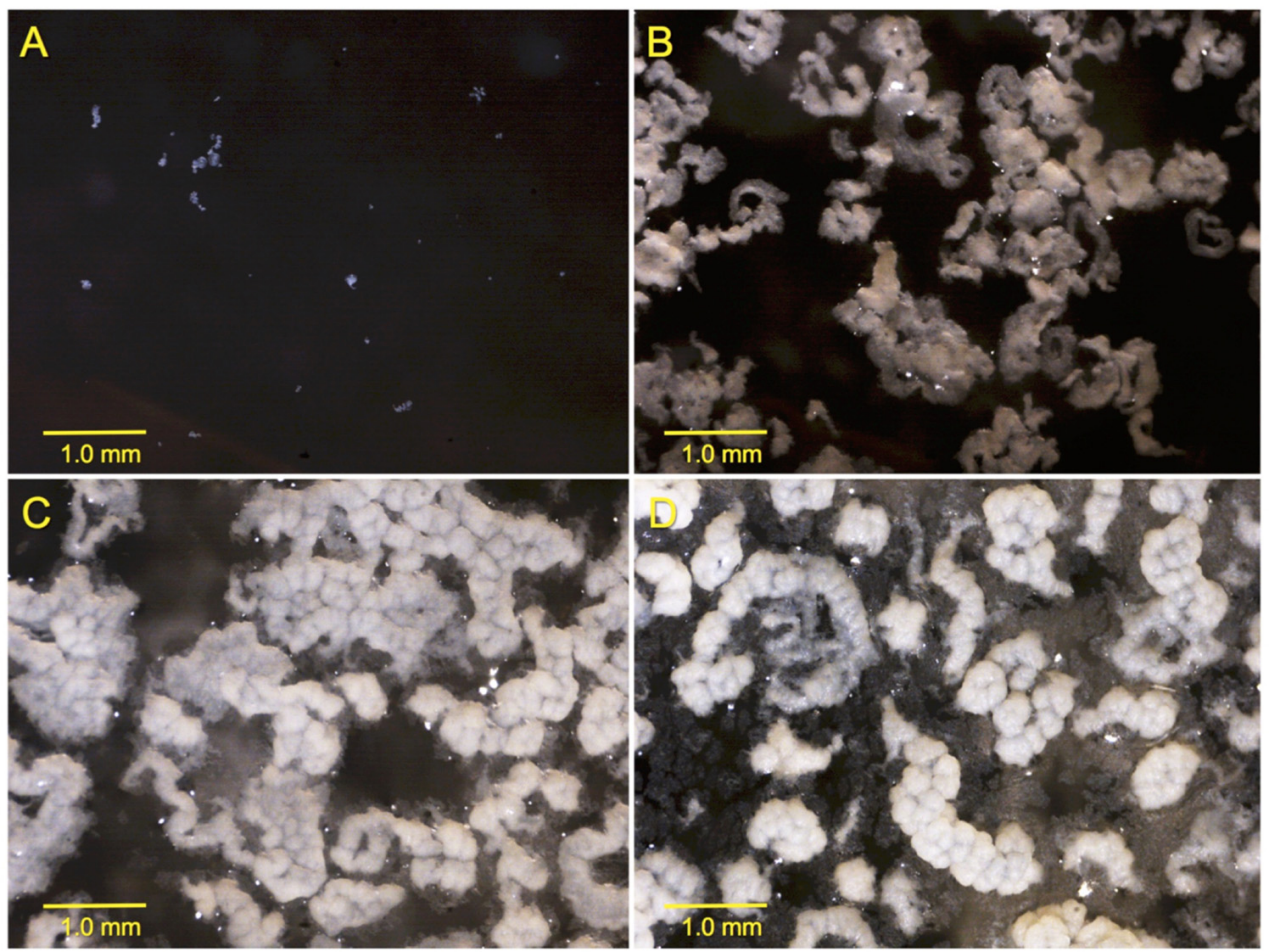
Kinetics of biofilm production by *M. bovis* BCG. Cultures of *M. bovis* BCG Pasteur are shown at different times: 24 h (**A**), 7 days (**B**), 10 days (**C**), and 14 days (**D**). Images were taken at 20X.

**Fig. 3:**
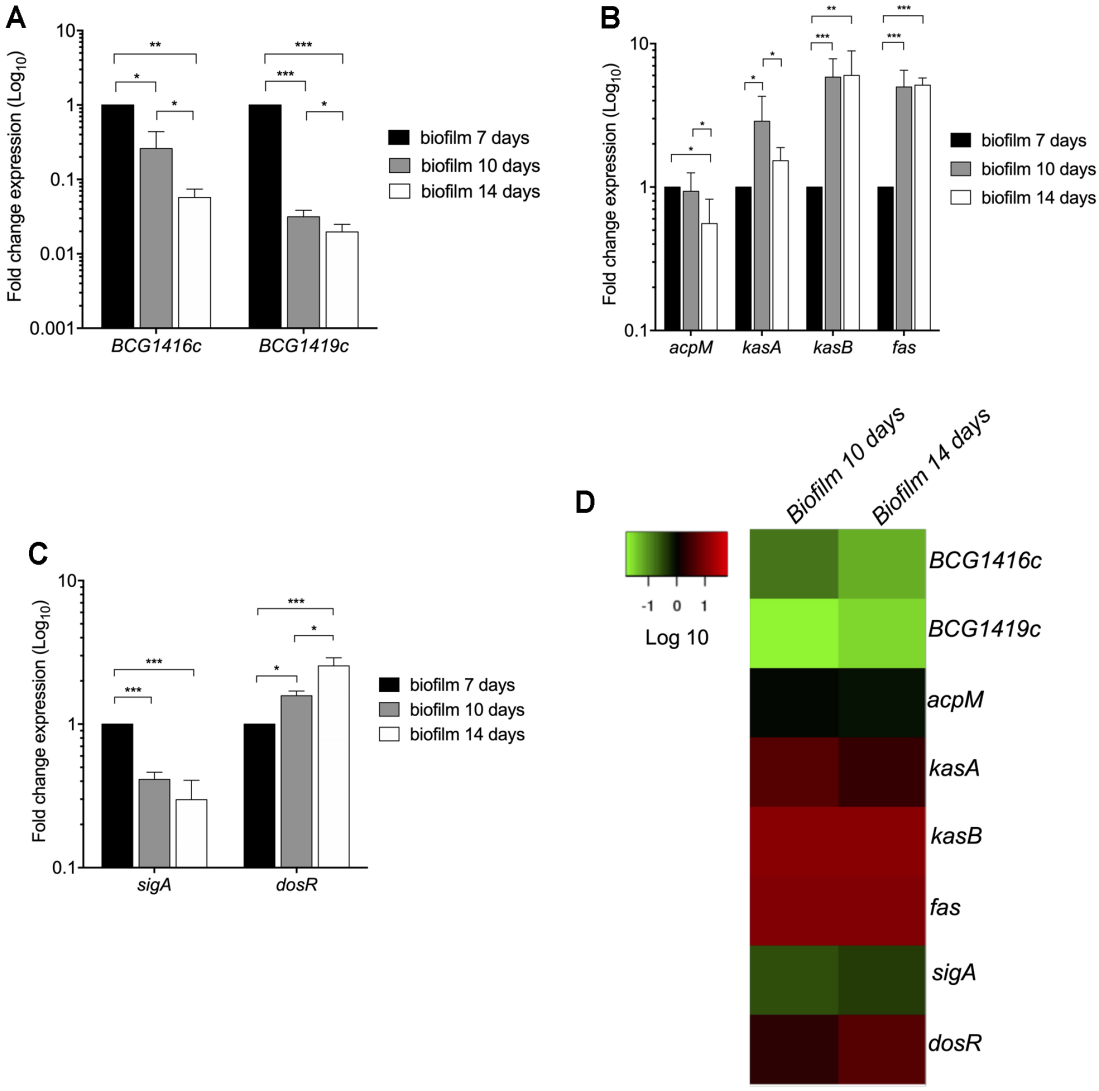
Expression of c-di-GMP, *dosR*, and mycolic acid metabolism-related genes of *M. bovis* BCG during biofilm formation. (**A**) Fold change expression (qRT-PCR) of *BCG1416c* and *BCG1419c* genes, (**B**) *acpM, kasA, kasB* and *fas* genes, and (**C**) *sigA* and *dosR* genes. Data represent the mean of two independent experiments performed in triplicates (mean ± SD). (**D**) Heatmap of gene expression levels expressed as fold change in a Log_10_ scale of biofilm formation at 7, 10, and 14 days relative to 7-days old cultures. The color-coding scale shows up-regulation in red and down-regulation in green. 16S rRNA was used as a reference gene for normalization. Statistically significant was considered as follows; ns, not significant, *p < 0.05, **p < 0.01, ***p < 0.001.

c-di-GMP synthesis and degradation, which are controlled by diguanylate cyclases (DGC) and phosphodiesterases (PDE), respectively, are important mechanisms for biofilm formation [20]. In BCG, we have already shown that either purified synthetic c-di-GMP, or deletion of the *BCG1419c* gene (encoding c-di-GMP PDE) increase biofilm production [8]. In that study, we determined that BCG forms fully mature biofilms after 2 weeks of growth in Sauton medium with no detergent, incubated at 37°C and 5% CO_2_. Therefore, we monitored growth on a daily basis and decided to take samples for qRT-PCR at 1, 7, 10, and 14 days of culture, to represent adaptation to the media (day 1), visible cell to cell attachment or intercellular aggregation (day 7), visible initial attachment to the plastic walls of the tissue culture flasks or substratum attachment (day 10), and mature biofilms (day 14) (Fig. 2). Because a structure leading to biofilm became visible after 7 days, we evaluated the expression of *BCG1416c* and *BCG1419c* at 7, 10, and 14 days, and used the first time point as a reference for the remaining steps. Compared to 7 days, the expression of *BCG1416c* decreased 3.8- and 17.5-fold during biofilm formation at 10 and 14 days, respectively (Fig. 3A). In terms of the expression of *BCG1419c*, it was 31.8- and 51-fold lower during biofilm formation at 10 and 14 days, respectively (Figs. 3A and D).

Regarding *acpM, kasA, kasB* and *fas* genes, which participate in the biosynthesis of MAs [21], the expression of such genes was mainly enhanced during biofilm formation (Figs. 3B and D). At the substratum attachment step (10 days), the expression of both *kasB* and *fas* was increased 6- and 5-fold, respectively, while at 14 days the expression did not change with respect to 10 days (Figs. 3B and D). The expression of *acpM* showed a slight decrease of 1.8-fold at 14 days with respect to 7 days. In these conditions, *sigA* transcription was lowered at 10 days (2.4-fold), whereas *dosR* expression was boosted at 10 and 14 days of biofilm formation as compared to 7 days, showing an increase in expression over time during biofilm production (Figs. 3C and D). It is worth noting that expression of *BCG1416c* and *BCG1419c* showed an opposite trend compared with *kasA*, *kasB*, and *fas* (Fig. 3), when cultures progressed from cell to cell attachment or intercellular aggregation (day 7) to substratum attachment to the plastic walls (day 10).

### Maturation of BCG Biofilms Modifies the Pattern of Mycolic Acids in a Time-Course Manner

In *M. smegmatis*, GroEL1, via its interaction with KasA, participates in shifting the MA pattern to produce an increased proportion of C_56_-C_58_ fatty acids and allow biofilm maturation [22]. In the same bacteria, it has been proposed that the decreased abundance of TDM observed during biofilm maturation in *M. smegmatis* was likely due to enzymatic hydrolysis [23]. Here, we observed that transcription of *kasA, kasB*, and *fas* was activated during the transition from intercellular aggregation (day 7) to visible attachment to the substrate (plastic walls) (day 10), with kasA transcription slightly decreasing in mature biofilms, while that of kasB and fas was sustained at this step (Fig. 3B).

**Fig. 4:**
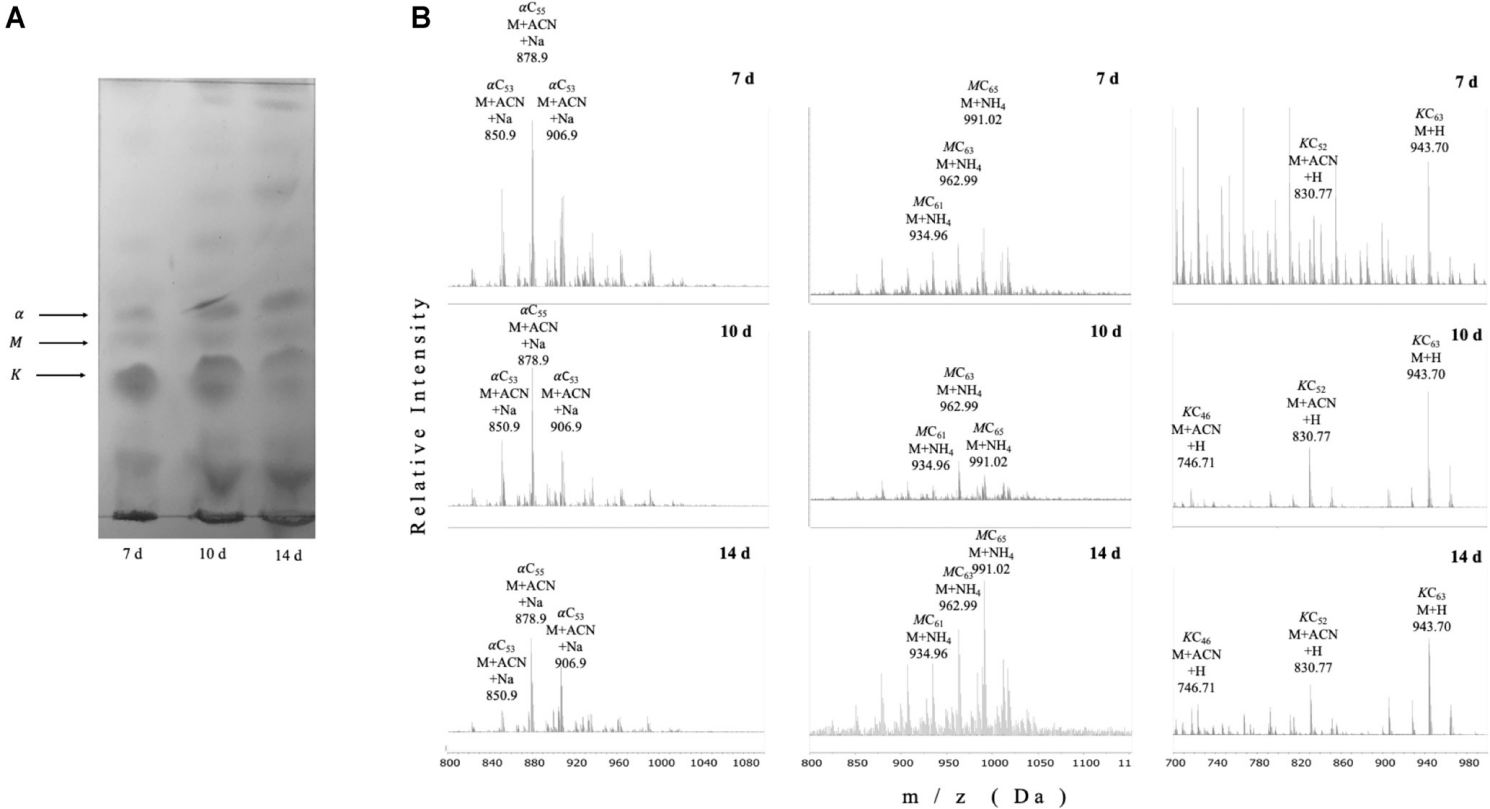
BCG reduces TDM production and changes mycolic acid methyl ester profiles during biofilm production. TDM was extracted from BCG biofilm cultures at the indicated time points (**A-B**) using petroleum ether, to later produce fatty and mycolic acid methyl esters, which were separated by TLC (**A**) or UHPLC followed by ESI-Q-TOF (**B**). The positions of alpha (α), methoxy (M), and keto (K) mycolates are indicated based on their reported migration in the solvent system used. In (**B**) the three most abundant peaks for Mycolic Acid Methyl Esters (MAMEs) according to the relative abundance (Y axis) with their corresponding m/z ratio (X axis) and formula are depicted. d stands for days of culture as surface pellicles/(biofilms). Times of retention during UHPLC were as follows: αCn, 25.2 s, MCn, 29.4 s, and KCn, 33.6 s. Lipids were extracted from two independent experiments using biological triplicates, with the same lipid pattern obtained in all instances.

We next evaluated the lipid profile of petroleum ether-extractable fractions from biofilm cultures at different steps during its production. The apparent relative abundance of alpha-, keto- and methoxy-mycolic acid methyl esters decreased during biofilm maturation in BCG (Fig. 4A). We detected by UHPLC-ESI-QTOF MS that most acyl chains were short (C_53_-C_65_) as it has been reported for *M. smegmatis* [22]. The relative proportion of the three most abundant MAs differed during biofilm maturation: α-mycolates with acyl chains in the range of C_53_-C_57_ decreased during biofilm maturation, while methoxy mycolates harboring C_61_-C_65_ acyl chains increased from day 10 to day 14, and the most abundant keto species (C_52_ and C_60_) remained practically stable. (Fig. 4B). The times or retention for each species in the UHPLC were αCn : 6.51 min, MCn : 7.07 min, and KCn : 3.4 min.

### C-di-GMP Modifies the Pattern of Mycolic Acids in a Concentration-Dependent Manner

**Fig. 5:**
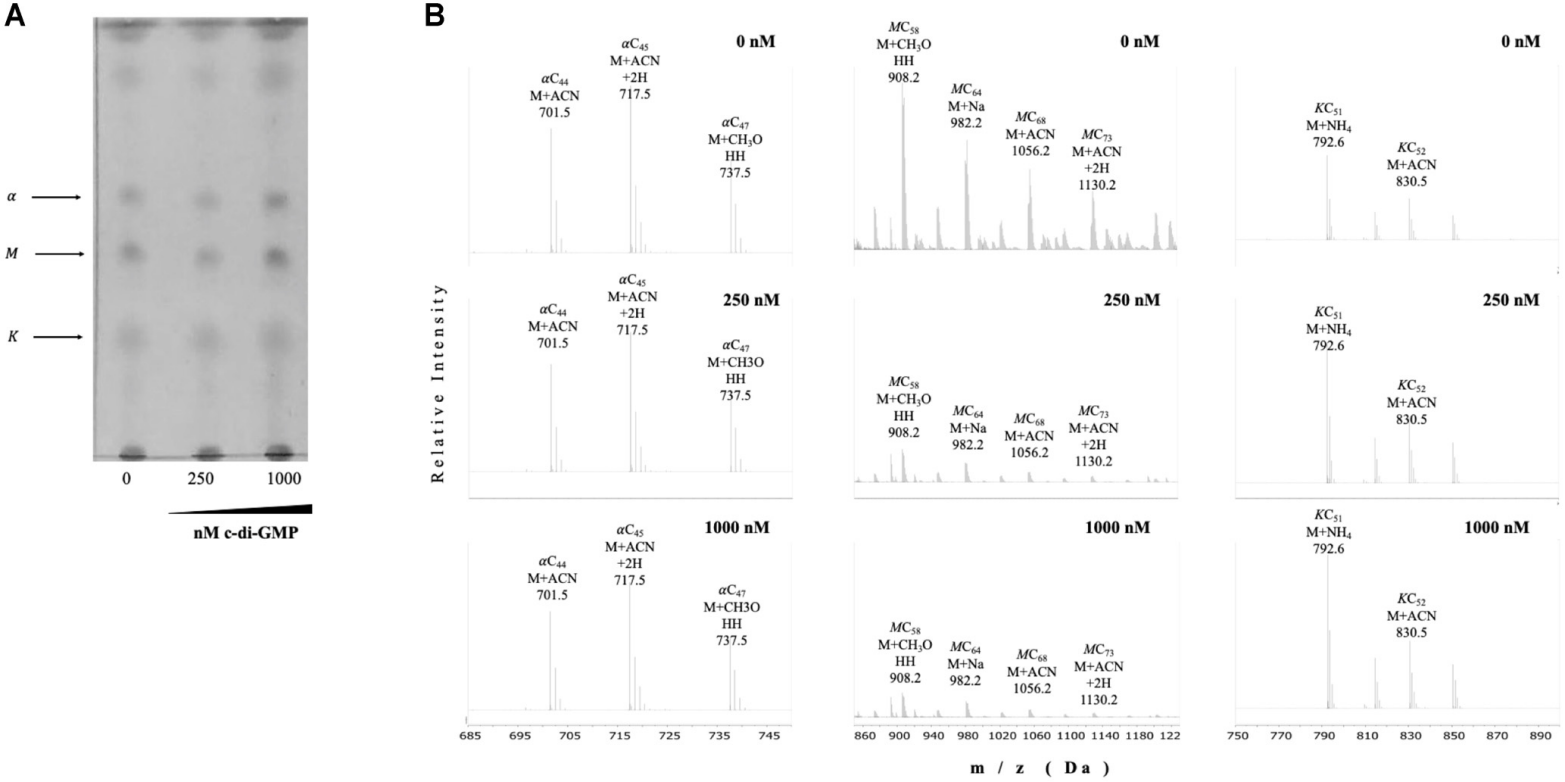
C-di-GMP modifies the pattern of mycolic acids in a concentration-dependent manner. TDM was extracted from BCG planktonic, shaken cultures with or without synthetic c-di-GMP at the indicated final concentrations, using petroleum ether, to later produce fatty and mycolic acid methyl esters, which were separated by TLC (**A**) or UHPLC followed by ESI-Q-TOF (**B**). In (**B**), the positions of alpha (α), methoxy (M), and keto (K) mycolates are indicated, and the most abundant peaks according to the relative abundance (Y axis) with their corresponding m/z ratio (X axis) and formula are depicted. Lipids were extracted from two independent experiments using biological triplicates, with the same lipid pattern obtained in all instances.

The apparent inverse pattern we observed between expression of genes involved in c-di-GMP metabolism and that of genes involved in MA metabolism during biofilm production prompted us to evaluate whether this second messenger had any direct effect on the end products measured as MAMEs. For this, we added synthetic c-di-GMP to mid-log phase, planktonic cultures of BCG, and separated MAMEs by TLC (Fig. 5A), and by UHPLC-ESI-QTOF MS (Fig. 5B). We observed that the relative proportion of the most abundant MAs differed in the presence of 250 or 1000 nM of c-di-GMP: α-mycolates with acyl chains in the range of C_44_-C_47_ were basically unaffected, while methoxy mycolates harboring C_58_, C_64_, C_68_, and C_73_ acyl chains decreased in the presence of c-di-GMP, and the most abundant keto species (C_51_ and C_52_) showed a slight increase in the presence of c-di-GMP (Fig. 5B).

## Discussion

*M. tuberculosis* forms surface pellicles and biofilms attached to the surface of plastic wells with a matrix composed mainly of mycolic acids (MAs), and it has been hypothesized that the capacity to produce biofilms in vitro may be linked to persistent infections inside the host [5, 9, 24, 25]. The ubiquitous second messenger c-di-GMP is essential to control the transition between planktonic and sessile bacteria in many species [20], and addition of synthetic c-di-GMP enhances biofilm production by *M. bovis* BCG Pasteur [8]. We also determined that this strain in our culture conditions (Sauton with no detergent, starting at OD600nm≈0.03) produced mature biofilms after 2 weeks of culture as opposed to the 4-5 weeks needed by *M. tuberculosis* H37Rv [9]. We considered that this difference could have two possible explanations: (1) the duplication of the *glpD2* gene in BCG, encoding glycerol-3-phosphate dehydrogenase [26], where in Sauton media with glycerol as sole carbon source allows for faster growth as compared to *M. tuberculosis*, and (2) a different starting inoculum; while Ohja *et al*. [9] used a 1:100 dilution of a saturated culture (with no specific OD or CFU load mentioned), here we used a culture from stationary phase BCG (OD_600nm_≈1.2) and inoculated Sauton media up to an OD600nm≈0.03, and therefore our dilution is close to 1:40. Other than this, the images reported by Ohja *et al*. [9] for *M. tuberculosis* mature biofilms are identical to those produced by BCG [8].

Despite the knowledge of *BCG1419c* being involved in biofilm production [8], and that deletion of this gene or the *BCG1416c* gene modifies host response in vitro and in vivo, including protection versus TB when used as vaccine in mice [27-30], there was no report regarding their expression in planktonic or biofilm cultures. Here, we observed that expression of both *BCG1416c*, and *BCG1419c* genes was repressed in the stationary growth phase compared to logarithmic phase, with transcription of *acpM* increasing, *fas* decreasing, and *kasA/kasB* without significant changes (Fig. 1). During biofilm production, transcription of both *BCG1416c* and *BCG1419c* was diminished at the substratum attachment and biofilm maturation steps (10 and 14 days), while *kasA, kasB*, and *fas*, increased (Fig. 3). Considering the relative transcript levels reported here, where both *BCG1416c* and *BCG1419c* were more reduced in biofilm cultures than in planktonic cells (Figs. 1 and 3), this would suggest that maintaining high levels of c-di-GMP might interfere in producing a mature biofilm, and that it should decrease once the cells underwent both intercellular aggregation and attached to the substrate. Furthermore, it shows that while stationary phase cultures result in lower transcription of *BCG1416c*, *BCG1419c*, and *fas*, with no effect on *kasA/kasB* as compared to mid-log phase bacteria, for biofilm production, the reduced transcription of *BCG1416c* and *BCG1419c* coincides with increased transcription of *kasA/kasB*, and *fas*, therefore suggesting that regulation of these genes is different for planktonic and biofilm-forming mycobacteria, in the latter suggesting that reduced c-di-GMP levels are required for increased production of MA to maturate biofilms.

In this work, we also studied the transcription of *acpM, kasA, kasB* and *fas* genes, which encode for enzymes involved in MA biosynthesis either in de novo synthesis or in elongation of the fatty acids [31]. The rationale of focusing on these genes is that MAs represent, as of today, the most extensively characterized molecule involved in biofilm production by mycobacteria [9, 22, 23, 32]. Whereas the fas gene codes for a type I fatty acid synthase that catalyzes the de novo synthesis of acyl-CoAs from acetyl-CoA using malonyl-CoA, *kasA* and *kasB* code for enzymes that catalyze the condensation of acyl-AcpM and malonyl-AcpM, with AcpM commuting the growing acyl chain between enzymes that catalyze the elongation process of fatty acids [33, 34].

We observed that *acpM* and *fas* showed an inverse expression pattern between planktonic (Fig. 1) and biofilm cultures (Fig. 3), where synthesis of acyl-CoAs was favored during biofilm maturation while this was reduced in planktonic, stationary phase bacteria. Another difference observed was that the acyl chain length might not be dramatically modified in planktonic cultures, as *kasA/kasB* transcription remained similar in exponential and stationary phase (Fig. 1), while these genes increased transcription during biofilm production (Fig. 3). Moreover, *kasA* increased during substratum attachment and reduced during biofilm maturation, whereas *kasB* increased and remained constant at the latter steps of biofilm production. The decrease observed of *sigA* transcription along with the increase of *dosR* transcription (Fig. 3), supports the notion that at least some part of the biofilm culture is undergoing oxygen limitation, as low aeration reduced *sigA* mRNA levels [35] while it activates *dosR* [3]. It is worth mentioning that sigA mRNA has been reported to be reduced during stationary phase [35], as opposed to what we observed in our cultures (Fig. 1C), so it could be that in our case bacteria were actually in the transition from late-log to stationary phase, as Manganelli, et al. considered as stationary phase when bacteria reached OD_600nm_ 2.8, whereas we harvested BCG at OD_600nm_ 1.2, in addition to using different conditions for incubation (roller bottles versus tissue culture vented-flasks shaken at 100 rpm, respectively).

As mentioned above, given that *kasA* increased during substratum attachment and reduced during biofilm maturation, whereas *kasB* increased and remained constant at the latter steps of biofilm production, these results suggest that shortening of mycolates to maturate biofilms requires decreased activity of KasA and/or sustained activity of KasB. In this regard, it has been found that in the fast-growing *M. smegmatis* strain, overexpression of *kasA* delays maturation of biofilms with no production of short-chain mycolates [22]. Moreover, in *M. tuberculosis*, rendering KasB inactive leads to shorter MAs, lack of acid-fast staining, and attenuation in mice [36], results which are in agreement with phenotypes observed upon deletion of *kasB* [37].

The transcriptional repression of *BCG1416c* and *BCG1419c* (Fig. 3A), coupled to induction of *fas, kasA*, and *kasB* (Fig. 3B), and the reduced expression of the latter 4 genes in biofilms produced by a *BCG1419c* mutant (encoding a c-di-GMP PDE)[30], may suggest that c-di-GMP participates in the reduction and modification of the MA pattern in BCG (Fig. 4). It is worth noting that deletion of the c-di-GMP-PDE encoding gene *BCG1419c* not only affected transcription of the genes mentioned above, but also expression of a number of proteins involved in an array of cellular functions [28], which provides support for the hypothetically expected rise in c-di-GMP occurring in this strain as compared with wild-type BCG, as it is known that second messengers can have multiple downstream targets, thereby expanding the scope of signal transmission integrated and relayed by them.

To dissect whether c-di-GMP has the hypothesized negative effect on MA, and that these changes were not the consequence of another confounding factor discovered during biofilm production (*e.g.*, nutrient or oxygen availability, presence of potentially toxic byproducts, inter or intracellular signaling differentially regulated, etc.), we added synthetic c-di-GMP to exponential phase cultures of BCG, and found that there was a reduction of methoxy mycolates, increase of keto species and practically no affect on α-mycolates (Fig. 5), therefore confirming the participation of this second messenger in modulating the fine structure of MA, by a mechanism that remains to be determined. In *M. smegmatis*, the transcriptional regulator LtmA had a positive role in controlling transcription of a number of genes involved in MA modification, but none of the genes homologous to those studied here (MSMEG_436, *acpM*; MSMEG_4327, *kasA*; MSMEG_4328, *kasB*; or MSMEG_4757, *fas*) was under the control of this regulator [38], therefore potentially ruling out that genes homologous to *M. smegmatis* LtmA are responsible for regulation of MA in BCG. On the other hand, c-di-GMP was shown to bind to EthR, and control expression of *ethA* in *M. tuberculosis* [39]. Interestingly, an *ethR* deletion mutant modified the production of C_24_ α-MA, and C_24_/C_26_ keto MA in BCG Pasteur in mid-log phase planktonic cells [40]. It might be worthwhile to determine whether *ethR* plays any role in biofilm production and regulation of MAs produced under this condition. Furthermore, post-transcriptional regulation could also be mediated via c-di-GMP as has been described for several bacteria in response to environmental cues such as nutrient availability or physiological stresses such as changes in osmolarity, pH, oxidative stress, or presence of antimicrobial compounds [41], although none of these has been proven to occur in mycobacteria yet. The only known conditions that induce transcription of *Rv1357c* (BCG1419c homologous gene in *M. tuberculosis* H37Rv) are intracellular growth within macrophages and the presence of arachidonic acids in cultures (TB database, http://genome.tbdb.org/cgi-bin/GeneDetails.html?id=SRv1357c) whereas H_2_O_2_ induced *Rv1354c* (BCG1416c homologous in gene in *M. tuberculosis* H37Rv, http://genome.tbdb.org/cgi-bin/GeneDetails.html?id=SRv1354c). Induction of *Rv1357c* within macrophages correlates with the reduced capacity of BCG mutants in *BCG1419c* to replicate in these cells as reported by us [28] and others [42].

We consider worth mentioning that changes in transcription of *kasA, kasB*, and *fas*, as well as changes in MA profiles during biofilm production (Figs. 3 and 4), are coincident with induction of a dormancy program as observed by *dosR* induction (Fig. 3). There is evidence that during in vitro dormancy, *M. tuberculosis* reduced its MA content, especially where most α- and keto-MA species reduced their abundance while methoxy MA increased during dormancy [43]. In our work, when we used petroleum ether to extract mostly TDM [16] from BCG Pasteur, we observed that for the most abundantly produced MA species, the relative abundance of α-mycolates decreased during biofilm maturation, with methoxy mycolates increasing from day 10 to day 14, while the most abundant species of keto-MA remained practically stable (Fig. 4B). The decrease in α-MA along with the increase in methoxy-MA during maturation of BCG biofilms are in agreement with their reported changes during dormancy in *M. tuberculosis* [43], therefore supporting the notion that dormancy and biofilms overlap, at least to some extent. On the other hand, under oxygen limitation, it was found that BCG Pasteur increased synthesis of α-MA with C_76_, C_78_, and C_80_ acyl chains while it reduced the amount of keto MA with C_82_, C_84_, and C_86_ acyl chains [44], species that are different (longer) than the ones most abundantly produced during biofilm conditions (Fig. 4).

Previous reports indicated that methoxy mycolates were not produced by BCG Pasteur [45, 46], a defect linked to a single-nucleotide nonsynonymous point mutation in *mmaA3* that causes a glycine-to-aspartic acid substitution at position 293 that impairs their production [47]. However, in this latter publication, the same BCG Pasteur strain used in our work, a faint but detectable spot is apparent on TLC plates, even in the absence of introduction of the *mmaA3* gene obtained from BCG Russia [47], leading us to wonder whether the particular substrain used in this report (ATCC 373534) is still able to produce trace amounts of methoxy mycolates. In support of this view, Shui and co-workers, using a similar approach (HPLC-ESI-QTOF) to the one employed in our work, reported that methoxy-MAs were very low abundant in BCG Pasteur, and very high in *M. tuberculosis* [44]. Similarly, low levels of methoxy mycolates have also been reported for BCG Danish and Glaxo strains, while BCG Tokyo produced levels similar to those found in *M. tuberculosis* H37Rv [48]. In addition, the lack of *mmaA4* resulted in loss of both methoxy- and keto-MA, while overexpression of *mmaA3* resulted in selective loss of keto-MA only [49]. These findings, coupled to the above mentioned possibility of c-di-GMP having a negative role in modulating *acpM, kasA, kasB*, and *fas* transcription, suggest that, even though we did not test the effect of c-di-GMP directly on transcription of *kasA, kasB*, and *fas* genes, it could be that synthetic c-di-GMP might affect keto-and methoxy-MA production via alteration of MmaA3 and/or MmaA4 activity, although this remains to be formally proven by testing a potential in vitro binding of c-di-GMP to MmaA3 and/or MmaA4.

We acknowledge that our work is limited because we have not been able to determine the levels of intracellular c-di-GMP in our experiments, which has remained elusive in our laboratory. Another limitation is the use of samples of total RNA extracted from whole biofilms, so we did not address at the single cell level the impact in BCG of being at a different spatial position within the biofilm, which may impact access to relevant environmental cues such as nutrients and oxygen. Nevertheless, changes detected in keto- and methoxy-MA upon addition of synthetic c-di-GMP confirm the participation of this second messenger in modifying MA profiles.

**Fig. 6:**
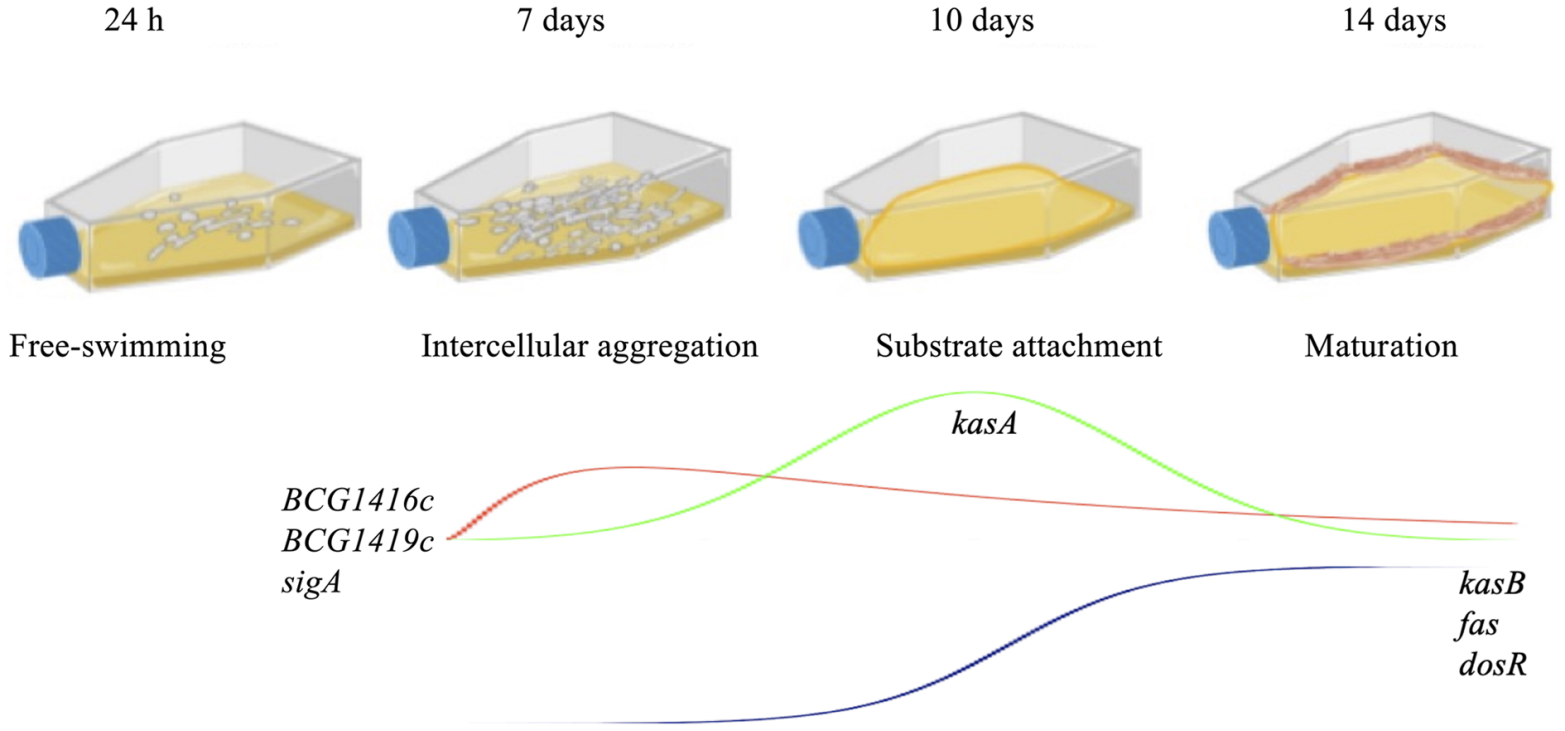
Schematic representation of the steps involved in biofilm production and temporal profiling of transcription from selected BCG genes. In our model, BCG starts as free-swimming bacteria (24 h) that in the absence of detergent, forms microcolonies of aggregated cells readily visible (7 days). Later, these aggregates attach to the plastic wells (10 days), to finally produce mature surface pellicles that cover all the air-liquid interphase as well as part of the plastic wells (14 days). Genes evaluated and their pattern of expression are indicated according to the values determined at the different phases of biofilm production.

Other genes have been found to be relevant for biofilm production in *M. tuberculosis*, such as *PE1, nirB, PPE5, mycP1*, and *pks1*, among others [50], although their temporal requirement during biofilm production has not been elucidated. We would like to mention that attachment to the microplate plastic well (day 10 in our model) may not necessarily represent a true biologically meaningful step for *M. tuberculosis*-complex bacteria. Nevertheless, considering the experimental approach used by us and others to follow biofilm production by mycobacteria [22, 23, 51], including *M. tuberculosis*, by means of culturing these bacteria in microplate wells or Petri dishes, to later monitor the capacity to produce or not produce biofilms [9, 32, 50, 52], may not be a relevant yet technically unavoidable step. In summary, as schematically represented in Fig. 6, our study constitutes the first evidence of a molecular intersection between dormancy and cell wall remodeling that occurs in biofilms and shows a differential effect of c-di-GMP on MA metabolism.

## Figures and Tables

**Table 1:** Primers and temperatures used for qPCR.

Primers	Sequence (5′ to 3′)	Temp (°C)	Source
*acpM-F*	ACAAGTACGGCGTCAAGATCC	58	This study
*acpM-R*	TTCTTCCTCGAGCTTCTGGAT		
*kasA-F*	TGAACTACGAGACACCCGATC	59	This study
*kasA-R*	AACCCGAACGAGTTGTTGAC		
*kasB-F*	ATGGAAGGAACGTTCGCAAG	59	This study
*kasB-R*	CAGCAACAACTTCCACGTAGTC		
*fas-F*	TCCAGACGCTATTCACCGATG	58	This study
*fas-R*	TCACGAAAAAGGGCACATCC		
*BCG1416c-F*	TCAAAGAAGTCGGCGTTCAC	59	This study
*BCG1416c-R*	TGCCGCACGAATGTTTTG		
*BCG1419c-F*	GAGAACAACGGACTGATGGTG	57	This study
*BCG1419c-R*	TTGACGCTGACAAACGGTT		
*sigA-F*	ATCGCGCGAAAAACCATCTG	59	This study
*sigA-R*	TGGTGTAGTCGAACTTCTCCAC		
*dosR-F*	ATGGCAACGGCATTGAACT	58	This study
*dosR-R*	AGAATCGCATCTAGCATGGC		
*rrs-F*	GTAATCGCAGATCAGCAACG	60	[53]
*rrs-R*	TTCGGGTGTTACCGACTTTC		

## References

[1] Houben RMGJ, Dodd PJ (2016). The global burden of latent tuberculosis infection: a re-estimation using mathematical modelling. PLoS Med..

[2] Menzies NA, Wolf E, Connors D, Bellerose M, Sbarra AN, Cohen T (2018). Progression from latent infection to active disease in dynamic tuberculosis transmission models: a systematic review of the validity of modelling assumptions. Lancet Infect. Dis.

[3] Voskuil MI, Schnappinger D, Visconti KC, Harrell MI, Dolganov GM, Sherman DR (2003). Inhibition of respiration by nitric oxide induces a *Mycobacterium tuberculosis* dormancy program. J. Exp. Med..

[4] Mehra S, Foreman TW, Didier PJ, Ahsan MH, Hudock TA, Kissee R (2015). The DosR regulon modulates adaptive immunity and is essential for *Mycobacterium tuberculosis* persistence. Am. J. Rrespir. Crit. Care Med..

[5] Flores-Valdez MA (2016). Vaccines directed against microorganisms or their products present during biofilm lifestyle: can we make a translation as a broad biological model to Tuberculosis? Front. Microbiol..

[6] Boyd CD, O'Toole GA (2012). Second messenger regulation of biofilm formation: breakthroughs in understanding c-di-GMP effector systems. Annu. Rev. Cell Dev. Biol..

[7] Gupta K, Kumar P, Chatterji D (2010). Identification, activity and disulfide connectivity of C-di-GMP regulating proteins in *Mycobacterium tuberculosis*. PLoS One.

[8] Flores-Valdez MA, Aceves-Sanchez Mde J, Pedroza-Roldan C, Vega-Dominguez PJ, Prado-Montes de Oca E, Bravo-Madrigal J (2015). The cyclic Di-GMP Phosphodiesterase gene Rv1357c/BCG1419c affects BCG Pellicle production and in vivo maintenance. IUBMB Life.

[9] Ojha AK, Baughn AD, Sambandan D, Hsu T, Trivelli X, Guerardel Y (2008). Growth of *Mycobacterium tuberculosis* biofilms containing free mycolic acids and harbouring drug-tolerant bacteria. Mol. Microbiol..

[10] Ojha AK, Jacobs WR Jr, Hatfull GF (2015). Genetic dissection of mycobacterial biofilms. Methods Mol. Biol..

[11] Esteban J, Garcia-Coca M (2017). Mycobacterium Biofilms. Front. Microbiol..

[12] Flores-Valdez MA, Segura-Cerda CA, Gaona-Bernal J (2018). Modulation of autophagy as a strategy for development of new vaccine candidates against tuberculosis. Mol. Immunol..

[13] Jahn CE, Charkowski AO, Willis DK (2008). Evaluation of isolation methods and RNA integrity for bacterial RNA quantitation. J. Microbiol. Methods.

[14] Aranda PS, LaJoie DM, Jorcyk CL (2012). Bleach gel: A simple agarose gel for analyzing RNA quality. Electrophoresis.

[15] Livak KJ, Schmittgen TD (2001). Analysis of relative gene expression data using real-time quantitative PCR and the 2(-delta delta C(T)) method. Methods.

[16] Arora R, Armitige L, Wanger A, Hunter RL, Hwang SA (2016). Association of pellicle growth morphological characteristics and clinical presentation of *Mycobacterium tuberculosis* isolates. Tuberculosis.

[17] Kremer L, Guerardel Y, Gurcha SS, Locht C, Besra GS (2002). Temperature-induced changes in the cell-wall components of *Mycobacterium* thermoresistibile. Microbiology.

[18] Hu Y, Coates AR (1999). Transcription of two sigma 70 homologue genes, sigA and sigB, in stationary-phase *Mycobacterium tuberculosis*. J. Bacteriol..

[19] Voskuil MI, Visconti KC, Schoolnik GK (2004). *Mycobacterium tuberculosis* gene expression during adaptation to stationary phase and low-oxygen dormancy. Tuberculosis.

[20] Jenal U, Reinders A, Lori C (2017). Cyclic di-GMP: second messenger extraordinaire. Nat. Rev. Microbiol..

[21] Bhatt A, Molle V, Besra GS, Jacobs WR, Kremer L (2007). The *Mycobacterium tuberculosis* FAS-II condensing enzymes: their role in mycolic acid biosynthesis, acid-fastness, pathogenesis and in future drug development. Mol. Microbiol..

[22] Ojha A, Anand M, Bhatt A, Kremer L, Jacobs WR, Hatfull GF (2005). GroEL1: a dedicated chaperone involved in mycolic acid biosynthesis during biofilm formation in mycobacteria. Cell.

[23] Ojha AK, Trivelli X, Guerardel Y, Kremer L, Hatfull GF (2010). Enzymatic hydrolysis of trehalose dimycolate releases free mycolic acids during mycobacterial growth in biofilms. J. Biol. Chem..

[24] Chen L, Wen YM (2011). The role of bacterial biofilm in persistent infections and control strategies. Int. J. Oral Sci..

[25] Orme IM (2013). A new unifying theory of the pathogenesis of tuberculosis. Tuberculosis.

[26] Brosch R, Gordon SV, Garnier T, Eiglmeier K, Frigui W, Valenti P (2007). Genome plasticity of BCG and impact on vaccine efficacy. Proc. Natl. Acad. Sci. USA.

[27] Pedroza-Roldan C, Guapillo C, Barrios-Payan J, Mata-Espinosa D, Aceves-Sanchez MD, Marquina-Castillo B (2016). The BCG Delta *BCG1419c* strain, which produces more pellicle in vitro, improves control of chronic tuberculosis in vivo. Vaccine.

[28] Segura-Cerda CA, Aceves-Sanchez MJ, Marquina-Castillo B, Mata-Espinoza D, Barrios-Payan J, Vega-Dominguez PJ (2018). Immune response elicited by two rBCG strains devoid of genes involved in c-di-GMP metabolism affect protection versus challenge with M. tuberculosis strains of different virulence. Vaccine.

[29] Segura-Cerda CA, Aceves-Sanchez MJ, Perez-Koldenkova V, Flores-Valdez MA (2019). Macrophage infection with combinations of BCG mutants reduces induction of TNF-alpha, IL-6, IL-1beta and increases IL-4. Tuberculosis (Edinb).

[30] Flores-Valdez MA, Pedroza-Roldan C, Aceves-Sanchez MJ, Peterson EJR, Baliga NS, Hernandez-Pando R (2018). The BCGDeltaBCG1419c vaccine candidate reduces lung pathology, IL-6, TNF-alpha, and IL-10 during chronic TB infection. Front. Microbiol..

[31] Zimhony O, Vilcheze C, Jacobs WR, Jr (2004). Characterization of Mycobacterium smegmatis expressing the *Mycobacterium tuberculosis* fatty acid synthase I (fas1) gene. J. Bacteriol..

[32] Wright CC, Hsu FF, Arnett E, Dunaj JL, Davidson PM, Pacheco SA (2017). The *Mycobacterium tuberculosis* MmpL11 cell wall lipid transporter is important for biofilm formation, intracellular growth, and nonreplicating persistence. Infect. Immunity..

[33] Schaeffer ML, Agnihotri G, Volker C, Kallender H, Brennan PJ, Lonsdale JT (2001). Purification and biochemical characterization of the *Mycobacterium tuberculosis* beta-ketoacyl-acyl carrier protein synthases KasA and KasB. J. Biol. Chem..

[34] Schaeffer ML, Agnihotri G, Kallender H, Brennan PJ, Lonsdale JT (2001). Expression, purification, and characterization of the *Mycobacterium tuberculosis* acyl carrier protein, AcpM. Biochim. Biophys. Acta.

[35] Manganelli R, Dubnau E, Tyagi S, Kramer FR, Smith I (1999). Differential expression of 10 sigma factor genes in *Mycobacterium tuberculosis*. Mol. Microbiol..

[36] Vilcheze C, Molle V, Carrere-Kremer S, Leiba J, Mourey L, Shenai S (2014). Phosphorylation of KasB regulates virulence and acid-fastness in *Mycobacterium tuberculosis*. PLoS Pathog..

[37] Bhatt A, Fujiwara N, Bhatt K, Gurcha SS, Kremer L, Chen B (2007). Deletion of kasB in *Mycobacterium tuberculosis* causes loss of acid-fastness and subclinical latent tuberculosis in immunocompetent mice. Proc. Natl. Acad. Sci. USA.

[38] Li W, He ZG (2012). LtmA, a novel cyclic di-GMP-responsive activator, broadly regulates the expression of lipid transport and metabolism genes in Mycobacterium smegmatis. Nucleic Acids Res..

[39] Zhang HN, Xu ZW, Jiang HW, Wu FL, He X, Liu Y (2017). Cyclic di-GMP regulates *Mycobacterium tuberculosis* resistance to ethionamide. Sci. Rep..

[40] Ang ML, Zainul Rahim SZ, Shui G, Dianiskova P, Madacki J, Lin W (2014). An ethA-ethR-deficient *Mycobacterium bovis* BCG mutant displays increased adherence to mammalian cells and greater persistence in vivo, which correlate with altered mycolic acid composition. Infect. Immun..

[41] Martinez LC, Vadyvaloo V (2014). Mechanisms of post-transcriptional gene regulation in bacterial biofilms. Front. Cell Infect. Microbiol..

[42] Stewart GR, Patel J, Robertson BD, Rae A, Young DB (2005). Mycobacterial mutants with defective control of phagosomal acidification. PLoS Pathog..

[43] Raghunandanan S, Jose L, Gopinath V, Kumar RA (2019). Comparative label-free lipidomic analysis of *Mycobacterium tuberculosis* during dormancy and reactivation. Sci. Rep..

[44] Shui G, Bendt AK, Pethe K, Dick T, Wenk MR (2007). Sensitive profiling of chemically diverse bioactive lipids. J. Lipid Res..

[45] Minnikin DE, Minnikin SM, Dobson G, Goodfellow M, Portaels F, van den Breen L (1983). Mycolic acid patterns of four vaccine strains of *Mycobacterium bovis* BCG. J. Gen. Microbiol..

[46] Minnikin DE, Parlett JH, Magnusson M, Ridell M, Lind A (1984). Mycolic acid patterns of representatives of *Mycobacterium bovis* BCG. J. Gen. Microbiol..

[47] Belley A, Alexander D, Di Pietrantonio T, Girard M, Jones J, Schurr E (2004). Impact of methoxymycolic acid production by *Mycobacterium bovis* BCG vaccines. Infect. Immun..

[48] Watanabe M, Aoyagi Y, Ridell M, Minnikin DE (2001). Separation and characterization of individual mycolic acids in representative mycobacteria. Microbiol-Sgm..

[49] Sambandan D, Dao DN, Weinrick BC, Vilcheze C, Gurcha SS, Ojha A (2013). Keto-mycolic acid-dependent pellicle formation confers tolerance to drug-sensitive *Mycobacterium tuberculosis*. mBio.

[50] Pang JM, Layre E, Sweet L, Sherrid A, Moody DB, Ojha A (2012). The polyketide Pks1 contributes to biofilm formation in *Mycobacterium tuberculosis*. J. Bacteriol..

[51] Ojha A, Hatfull GF (2007). The role of iron in Mycobacterium smegmatis biofilm formation: the exochelin siderophore is essential in limiting iron conditions for biofilm formation but not for planktonic growth. Mol. Microbiol..

[52] Flores-Valdez MA, de Jesus Aceves-Sanchez M, Pedroza-Roldan C, Vega-Dominguez PJ, Prado-Montes de Oca E, Bravo-Madrigal J (2015). The cyclic di-GMP phosphodiesterase gene Rv1357c/BCG1419c affects BCG Pellicle production and in vivo maintenance. IUBMB Life.

[53] Ares MA, Rios-Sarabia N, De la Cruz MA, Rivera-Gutierrez S, Garcia-Morales L, Leon-Solis L (2017). The sigma factor SigD of *Mycobacterium tuberculosis* putatively enhances gene expression of the septum site determining protein under stressful environments. New Microbiol..

